# Effectiveness of nursing intervention session on health locus of control and self efficacy for women with preeclampsia

**DOI:** 10.1186/s12884-025-07447-w

**Published:** 2025-04-23

**Authors:** Nor El-Hoda Mohamed El-Sayed ElShabory, Reda Abdallah Abdel-Aziz Abdel-Gawad, Hanan Salem Sanad Mohammed, Azhar Abdel-Fatah Mohamed Shehata, Esraa Mostafa Abd El-Aty Ibrahim

**Affiliations:** 1 Maternity, Gynecology and Obstetrics Nursing, Faculty of Nursing, Port-Said University, Port-Said, Egypt; 2 Psychiatric and Mental Health Nursing, Faculty of Nursing, Minia University, Minia, Egypt; 3 Community Health Nursing, Faculty of Nursing, Zagazig University, Zagazig, Egypt

**Keywords:** Health locus of control, Nursing intervention, Preeclampsia, Self efficacy

## Abstract

**Background:**

Unfavorable fetal, neonatal, and maternal outcomes are more likely to occur in pregnancies complicated by hypertension. The degree of awareness regarding health circumstances and locus of control on health is a highly significant component in determining the effectiveness of adherence to therapy, which is influenced by determinant factors.

**Aim:**

Assess the effectiveness of nursing intervention session on health locus of control and self efficacy for women with preeclampsia.

**Method:**

Two group quasi-experimental study design was used in outpatient units of all Port Said City obstetric hospitals that adhere to the comprehensive health insurance. A purposive non-probability sample of one hundred and fifty pregnant women. A structured self administered questionnaire, the multidimensional health locus of control scale and general self efficacy scale.

**Results:**

Following nursing intervention sessions, pregnant women with preeclampsia in study group obtain higher mean score of internal, external powerful others of locus control on health and self efficacy (22.373 ± 9.316, 24.866 ± 7.323 & 15.640 ± 5.116) compared to the control group (20.866 ± 9.969, 23.640 ± 8.105 &12.080 ± 6.803) who didn’t not take part in nurse intervention sessions, with a statistically significant difference (*p* = 0.000) between the groups under study. Moreover, the study group’s external chance of health locus control mean scores were greater (21.013 ± 9.047) than the control group (19.946 ± 10.628) with none a noticeable difference between the groups under study.

**Conclusion:**

In women with preeclampsia, nursing intervention can have a positive impact on all aspects of locus of control over health and self efficacy.

**Trial registration number:**

The study protocol was registered by the Research Ethics Committee of the Faculty of Nursing, Port Said University with code number: NUR27on 9/7/2023.

## Background

Preeclampsia, eclampsia, persistent or chronic hypertension, and gestational hypertension are among the illnesses that fall under the broader category of prenatal hypertensive disorders. A persistent increase in blood pressure to 140/90 mm Hg or greater at least twice, separated by four hours, following the twenty week of pregnancy is known as gestational hypertension, an uncomfortable and stressful pathological condition [1]. In both developed and developing nations, it is a significant public health issue that raises the risk of perinatal death. 4.2% of Egyptian women are impacted and accounts for 18% of maternal fatalities worldwide [2]. Furthermore, the WHO [3] reports that there are 34% maternal deaths for every 100,000 births that are live between 2000 and 2020.

Increased the immediate and potential long-term hazards of mother-fetal issues are linked to gestational hypertension. This includes twice as high a risk of cardiovascular death and major adverse cardiovascular events, a two to four times greater chance of persistently elevated blood pressure, and a mother’s 1.5-fold elevated risk of stroke. There are risks for the embryo, including placental separation, oligohydramniosis, iatrogenic preterm birth, intrauterine growth retardation, and intrauterine foetal death [4].

For a pregnant woman to maintain consistent blood pressure control, she must adhere to the anti-hypertensive regimen. This program consists of a variety of behavioral and lifestyle adjustments including blood pressure monitoring, eating a healthy diet, exercising frequently, and taking prescription drugs as directed. When it comes to goal-setting, treatment planning, and regimen execution, adherence is all about choice and mutuality. On the other hand, adherence shows that the woman and doctor work together for enhancement the women health by incorporating the women values, lifestyle, and treatment choices with the doctor’s medical advice. This suggests that the medical regimen and treatment goals are cooperatively established by patients and physicians. They also keep using a prescription drug as directed [5].

According to scientific definitions, adherence is the extent whereby a patient’s free will behavior complies with medical professionals’ therapeutic suggestions. Patients who adhere to their treatment plan demonstrate that they are independent persons who take an active and voluntary part in setting and completing treatment goals. It has been observed that adherence to prescribed lifestyle modifications is as low as 10%, but the average degree of adherence to drug regimens is 50% [6]. The research by Sun et al. [7] indicates that it’s critical to forecast psychological aspects and aperson’s desire to achieve treatment goals in order to enhance antihypertensive medication adherence. These factors could be things like one’s own locus of control on health orientation, health attitudes, sociodemographic traits, illness knowledge, and regimen complexity.

Rotter is credited with developing the health locus of control hypothesis [8]. The phrase “locus of control” refers to how someone feels they have control over their lives. Actually, Rotter’s locus of control on health scale was the first to determine control orientation—the degree to which one’s behaviors contribute to achieving goals. The internal and external locus of control make up the health locus of control. A person with an internal locus of control believes that the things they do directly affect the results. An exterior center of control person thinks how events are the product of fate or strong external figures, like doctors [9].

Thus, locus of control is explained by Gerçek & Özveren [10] as a broad expectancy that can be applied when a person hasn’t possessed sufficient experience with a specific action or task to form certain expectancies. This is in line with Social Learning Theory. Thus, the locus of control is acceptable in more general or special circumstances such gestational diabetes, eclampsia, and preeclampsia. The health locus of control concept has been used in several studies to assess adherence to treatment plans.

Additionally, self efficacy is likely one of the most important factors influencing patients’ ability to manage their hypertension and conduct self-monitoring [11]. Self-efficacy is one of the cornerstones of Bandura’s social cognitive theory, which discusses how behavioral, environmental, and personal variables interact to cause both health and illness [12]. Furthermore, self efficacy is a useful tool for predicting, recognizing, and encouraging patients to take care of themselves while managing hypertension. Making lifestyle changes, such as changing one’s diet, quitting smoking, and exercising, requires a greater level of self efficacy and confidence [13]. According to [14], a key component of successful self-management of chronic illnesses is an elevated degree of self efficacy.

These selfcare tasks require a greater level of self efficacy to be completed. Self efficacy plays a major role in improving lifestyle modifications and hypertension control in preeclamptic women, as well as successful maintenance of healthy habits like exercise, dieting, and dietary changes [15].

Insuring commitment to healthy lifestyle habits and practices is a major responsibility of nurses. In order to lower maternal and newborn problems among women with preeclampsia, they can include health education, planning and goal-setting, role-modeling, and mastery experiences, as well as motivating messaging. Furthermore, the management of these women is positively impacted by the nursing intervention, which includes self-monitoring blood pressure, doing regular exercise and eating a healthy diet, and taking prescribed medicine [16].

### Significance of the study

Suhartono et al. [2] estimate that preeclampsia causes between 62,000 and 77,000 maternal fatalities annually, or around 18% of all maternal deaths globally. In contrast, it complicates 4.2% of pregnancies in Egypt. Following health care techniques for managing or preventing complications are widely recognized, most maternal mortality are preventable [3].

Individual perspectives about health, illness, and health care are significant because they influence a person’s self-care practices and care-seeking behavior. Proper self-care in the context of adhering to an antihypertensive routine will reduce the fatal complications and death of mothers and their fetus [9]. Therefore, it’s critical to comprehend the feeling of self efficacy and locus of control in preeclamptic women, since these psychological constructs are thought to be essential underlying factors that may affect how women behave and deal with their emotions as well as their overall health. It’s also critical that medical professionals understand that pregnancy is an opportunity to introduce health education that promotes health, prevents dangers, and manages difficulties from a long term and lifetime perspectives for the mother and her unborn child [17].

While holding an internal health locus of control is linked to better adherence to self-care regimens than external H LC, according to some researchers. Contrary findings have been found by others, so this isn’t always the case. Among hypertension women with elevated IHLC, poor adherence to antihypertensive medication has been seen [17]. There is actually insufficient research on health attitudes, such as health locus of control and adherence to antihypertensive medication among pre-eclamptic women. Therefore, this study was conducted to determine the effectiveness of nursing intervention session on health locus of control and self efficacy for women with preeclampsia.

### Aim of study

This study aimed to assess the effectiveness of nursing intervention session on health locus of control and self efficacy for women with preeclampsia.

It accomplished this by achieving the following goals:

- Assess HLOC (internal, external powerful others and external chance health locus of control) in women with preeclampsia before and after nursing intervention about preeclampsia.
- Assess self efficacy in women with preeclampsia before and following nursing intervention about preeclampsia.
- Design of an nursing intervention session about preeclampsia HLOC and self-efficacy for women with preeclampsia.
- Implement nursing intervention session about preeclampsia HLOC and self-efficacy for women with preeclampsia.
- Evaluate the effect of an nursing intervention session about preeclampsia on HLOC and self-efficacy for women with preeclampsia.

#### Research hypotheses

- Hypotheses I: Preeclamptic women who participate in nursing intervention sessions will have score higher than the control group on HLOC (internal, external powerful others, and external chance health locus of control).
- Hypotheses II: Preeclamptic women who participate in nursing intervention sessions will have self efficacy score higher than the control group.

### Operational definitions

#### Locus of control

It refers to how much people think there is a connection between their activities and the outcomes they achieve.

##### Internal locus of control

The idea that particular one has some degree of influence over one’s own life circumstances.

##### External locus of control

It relates to a belief that circumstances or forces outside of oneself—such as fate, chance, luck, influential people, or powerful others—determine one’s fate.

#### Self efficacy

Self efficacy is a factor that predicts health behavior in terms of women’s engagement in physical activity, weight loss, chronic illness prevention, and smoking cessation.

## Method

### Research design

To accomplish its stated goal, this study used a two-group quasi-experimental design (control and study groups with pre- and post-tests).

### Study setting

The present study was conducted in the outpatient departments of both two Port Said City obstetric hospitals that accept comprehensive health insurance: Dar Sahet ElmarAa Hospital and Alhayaa Hospital.

### Research subjects

The study used a purposeful non-probability sample of preeclamptic pregnant women.

### Sample size

The sample size was calculated using Epi-info 7 software tools, with an 85% level of confidence, a 5% margin of error, and a 10% prevalence of preeclamptic women according to Pieczykolan et al. [17]. The sample size estimated according to the previously stated criteria was 75 women. A total sample size of 150 pregnant women was planned for comparison between two groups.

### Inclusion criteria

The requirements for inclusion comprised. Aged between 20 and 35, the pregnant woman has had mild to moderate preeclampsia for at least two months (between 24 and 28 weeks of pregnancy). Free from other illnesses and willing to take part in the research.

* Pregnant women between weeks 24 and 28 are chosen because this is thought to be a crucial time frame for tracking preeclampsia cases. Preeclampsia symptoms are more noticeable at this point, which facilitates identification and the right kind of treatment. Additionally, beginning the intervention at this time provides enough time to evaluate the impact of the nursing intervention on self-efficacy and health locus of control, while ensuring follow-up until delivery to gain an accurate understanding of the results.

### Exclusion criteria

Women who had any other medical health issues during the study period were not included.

Prior to participating in the study, participants volunteered. In order to randomly allocate groups of women in various age and educational categories to either the intervention or control group, the women were divided into two groups. This approach increases the possibility of finding group differences.

### Tools of data collection

#### Three tools were used to collect the data

##### Tool I: a structured self-administered questionnaire

A standardized, self-administered questionnaire was used to gather general participant characteristics. It was created by the researchers in Arabic and is separated into the following two parts:

*The Socio-demographic data history (Part I)*: This included the age, education, occupation, place of residence, the crowding index, and family income of the women.

*Obstetric history (Part II)*: It included details on the frequency of abortions, gravida, and paras, and weeks of gestation.

##### Tool II: the multidimensional health locus of control scale (MHLC)

The English version was adapted from Khademi, Kaveh, Nazari, and Asadollahi [18], and the researchers adjusted it to fit Egyptian culture. Women suffering from preeclampia are measured according to several characteristics of HLC using this scale. The instrument comprises three subscales, including Locus of Control on Internal Health (IHLC), Health Locus of Control over External Powerful Others (EPHLC), and Health Locus of Control on External Chance (ECHLC), each with six items.

*Scoring System*

Using a Likert scale of 1 to 6 to indicate “strongly disagree” and “strongly agree,” is used to score the responses. Each subscale has a possible score range of 6 to 36. Respondents were categorized based on the subscale scores for which they had the largest predisposition to believe in the controlling source, i.e., the greater the score for locus of control.

##### Tool III: general self efficacy scale

It was adopted to evaluate the self efficacy of preeclamptic women. This English-language scale was taken from Galiana, Sánchez-Ruiz, Gómez-Salgado, Larkin, and Sansó [19]. The GSES consists of ten statements, such as: I’m sure I could handle unforeseen situations well; I know how to handle unforeseen situations because of my resourcefulness; If I work hard enough, I can always find a solution to a challenging problem; if someone disagrees with me, I can find a method to accomplish my goals; and if I make the required effort, I can usually solve most challenges.

*Scoring system*

The tool alternatives were ranked from completely incorrect (1) to perfectly correct (4). Higher degree indicate greater competency in handling everyday problems. Scores range from 4 to 40.

### Tool content validity

Six obstetricians and gynecologists from Port-Said University’s medical and nursing faculties examined the data collection tools the researcher had created. Evaluating the instruments’ relevance, significance, range, and lucidity was the aim of the assessment. The required modifications were put into place after they were consulted regarding the structure, organization, and coherence of the instrument.

### Content reliability

The coefficient of Cronbach’s alpha was computed in order to verify the reliability. Results showed that the locus of control on health scale was 0.86 also the overall scale of self-efficacy (GSE) was 0.76 [20].

### Ethical considerations

The study’s conduct was officially accepted and acknowledged by the Port Said University nursing faculty’s ethics committee, which went under code NUR 9/7/2023(27) based on its standards and the declaration of Helinski. Additionally, the chief manager of the aforementioned sitting accepted the study when it received the necessary clearances. Every woman in the study’s sample was made aware of her choice to participate or not. Every female participant in the sample received an explanation from the researcher regarding the aim of the study. They were told that all research data would be kept private and would only be utilized to further the goals of the inquiry. The privacy of the subjects was always respected. The women had to turn in written consent forms before they could be registered.

### Administrative design

The Dean of the Nursing Faculty and the Directorate of Health provided formal consent to the directors of the aforementioned meeting in the city of Port Said so that they may acquire authorization before beginning the study. They were informed that their information would only be utilized for research and had the choice to decline participation.

### A pilot study

16 randomly chosen woman from the previously stated sitting made up 10% of the study participants for the pilot research (8 women were allocated to the study group and 8 to the control group). This was done for estimating the time needed to complete the questionnaire and to evaluate the tool’s usefulness, clarity, and relevance. The questionnaire was changed, with some questions added and others removed, particularly in the sections that addressed women’s knowledge and habits. As a result, the female participants were excluded from the study’s overall sample in the pilot assessment. It didn’t require any modifications.

### Field work

The study’s data collection period ran from September 15, 2023, to December 31, 2023.

### Procedures

The process consists of three stages: preparation for the intervention, implementation of the intervention, follow up and outcomes evaluation.

#### Phase 1: preparation for the intervention

**Fig. 1:**
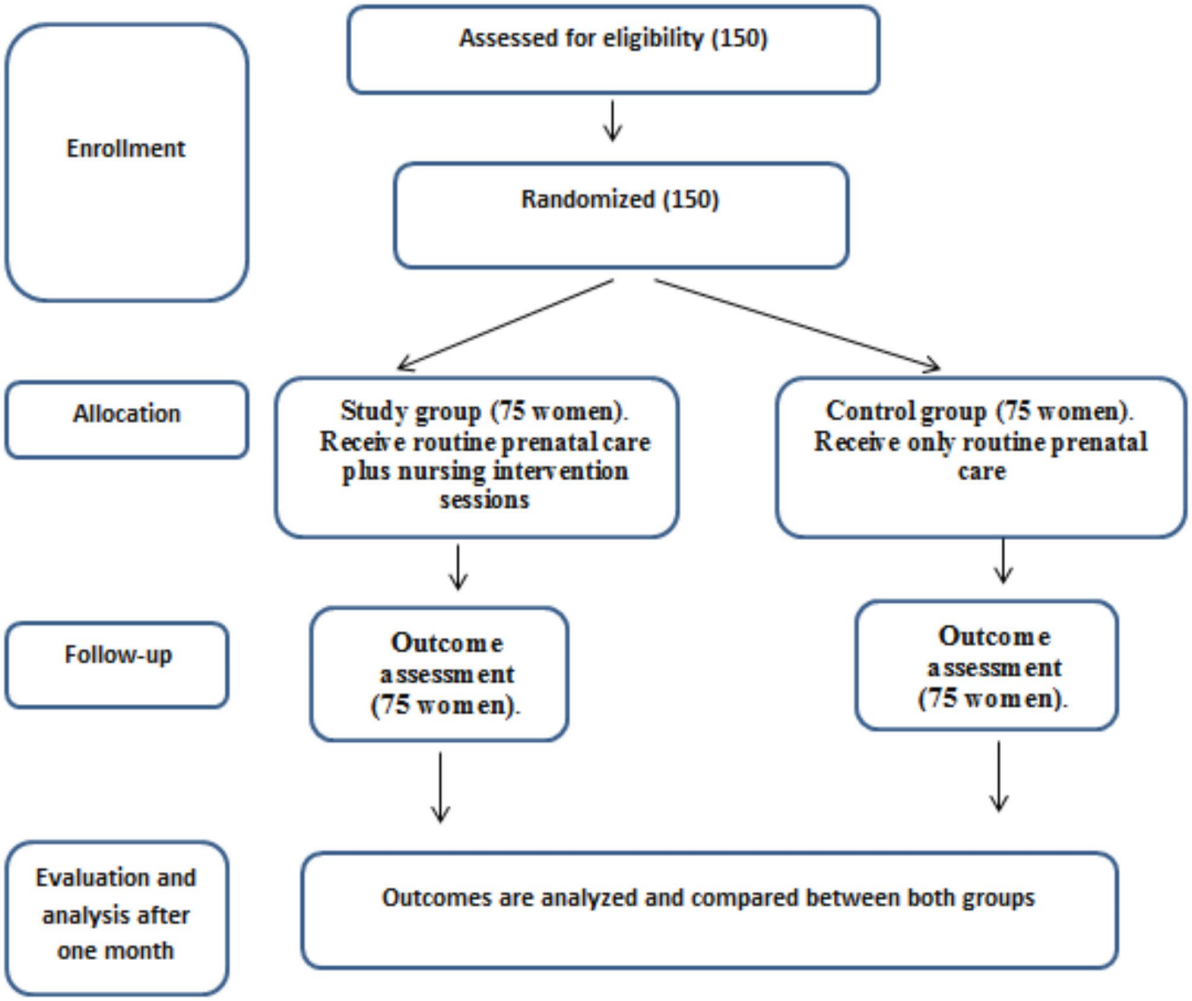
The consort flow diagram shows the study’s random assignment and participation of women

A total of 150 pregnant women were planned for comparison between two groups (Fig. 1). The creation of the intervention subgroups randomly took place during this phase. The goal of the training was to enable women to learn about preeclampsia and put that knowledge into practice. The following subjects were discussed: taking prescribed medication as directed, maintaining a healthy diet, and self-monitoring blood pressure. In order to address the participants’ initial worries and assist them in recognizing important issues during the sessions, each lady in the study group also attended a private face-to-face meeting on the same day, thirty minutes before the sessions. The standard routine clinic intervention sessions were given to the ladies who were part of the control group.

Creation of subgroups for the nursing intervention sessions: The researcher described the goals and methodology of the research to each eligible preeclamptic woman and her female companion during the first prenatal care visit (each woman was permitted to accompany single friend). It was essential that the friend possess prior knowledge about preeclampsia. This is to guarantee engaged engagement in group conversations (not required). After obtaining both groups’ informed written consents, each prospective participant was placed in a subgroup for nurse intervention sessions. Because learning in small groups of eight was more effective than learning in a big group of seventy-five, each subgroup was made up randomly of eight preeclamptic women. The primary intervention group consisted of ten subgroups.

For the study group of women, the intervention consisted of many preeclampsia nursing sessions. Eight women participated in each subgroup, the nursing sessions were held one day per week, which lasted four hours per day and were divided into two sessions: a two-hour theoretical session followed by a two-hour practical session. Group education was utilized for the intervention as it is more cost-effective than one-on-one instruction and provides an efficient approach for the areas it covers. The study setting’s quiet room was chosen for the participants’ convenience during the scheduling of the sessions. It was requested of all members of the same group that they attend future visits together. It was not required for the pals to attend.

#### Phase 2: implementation of the intervention

Using organized group sessions, nursing intervention sessions were delivered as part of the implementation. Each session proceeded according to a set order. Preeclampsia diagnosis and risk assessment preceded teaching and skill development in each session. The following were the key issues covered in nursing intervention sessions:

- Basic details regarding pregnancy-induced hypertension, including its definition, etiology, risk factors, types, and preeclampsia risk signs.
- Preeclampsia-related problems affecting mothers and newborns.
- Identification and management of preeclampsia.
- Locus of control on health: concept, strategies, and dimensions.
- Prevention of preeclampsia which attributed to a healthy self concept and a locus of control on health issues.
- Self-efficacy concepts and kinds.
- A variety of self-efficacy techniques and resources (such as goal-setting plans, motivational messaging, role modeling, and mastery experiences) strengthen their belief that they are in control of their condition.
- Teach women how to check their blood pressure on their own (this is the practical aspect).

Preeclampsia risk assessment diagnosis: All participants in the group underwent a physical examination at a single clinic within the first thirty to forty minutes for each educational session. A researcher completed the physical assessment. Members of the group were then instructed to proceed to a neighboring room to finish the instruction and skill-building of the session. Under the observation of the session researcher, women with preeclampsia actively participated in their care in subsequent sessions.

Preeclampsia nursing intervention sessions and skill development were used to deliver education. In order to help participants enhance their skills, a researcher would spend about five to ten minutes for each participant—demonstrating and allowing the individuals to repeat key skills. The goal of skill development was to educate the participants on blood pressure self-monitoring.

In the context of a group setting, each subgroup member is encouraged to participate in social support and group discussions with experienced guest speakers who have experience with preeclampsia, such as female friends. They were led by the researcher. Talks centered on exchanging other people’s experiences. This type of conversation regarding group members’ concerns about preeclampsia was allotted thirty minutes. In the subgroup meetings and conversations, the participant is free to interact with and meet the other participants when she went to the next follow-up visit at the high-risk outpatient clinic. This concept made it possible for information to grow organically. The sessions took place weekly on Sundays and Tuesdays for five weeks.

#### Phase 3: follow up and outcomes evaluation

Phone numbers of study participants were gathered for future correspondence during the study period. The first appointment started between 24 and 28 weeks of pregnancy and continued until delivery. Subsequent visits were scheduled every week or every two weeks, depending on the gestational age.

The intervention’s effects on women’s HLOC and self efficacy in preeclamptic women were evaluated. The first results were assessed at baseline, which was prior to the nursing intervention, and one month later. The women were asked to return to the study location so they could fill out questionnaires for the post-intervention assessment. Telephone calls were made to the participants who did not respond.

Individual prenatal routine care was provided as usual to the control group. Every individual was examined for a maximum of five to ten minutes, during which time their HLOC and level of self-efficacy were evaluated. The initial outcomes were evaluated using the baseline of the research study and after one month of individualized prenatal care. The participants would come back to the research area one month later to complete questionnaires. We followed up with the absentee participants over the phone. Plans to address the concerns of skill development and education were not scheduled.

### Data analysis

Version 20.0 of all statistical analyses were performed using the Statistical Package for Social Science for Windows (SPSS). The mean ± standard deviation was used to show the continuous data (SD) and were regularly distributed. Numerical and percentage representations of categorical data. Student’s t-test was utilized to determine the comparisons for variables that have ongoing information, while the Chi-square test was employed for variables with data categorized by type. The acceptable level for statistical significance was set at *p* ≤ 0.05.

## Results

Table 1 shows frequency and percentage distribution of the studied groups regarding personal characteristics. In terms of age, the highest percentage of study participants in the control group (57.0%) was between the ages of 30 and 35. Despite the fact that about 74% of the study’s participants were under 29, the two groups’ differences were statistically significant (p 0.036).

In terms educational achievement went, equivalent of half the women in studied groups were diploma holders. With a household income of more than 3000EP, housewives made up the largest percentage of mothers in both groups. Regarding the count of family members, both groups’ mean crowding scores were 1.4 ± 0.3. With the exception of age, there were no differences in significance between the studied groups’ work status, education level, monthly family income, or the crowding index.

Based on their obstetric history, Table 2 displays the proportion of the women under examination in the studied groups. About half of the women in the study group (51%) have one to two number of gravida, with a mean score of 2.4 ± 1.1 pregnancies, compared to about half of them were primigravida, with a mean score of 1.8 ± 0.5 in the control groups. Additionally, among 51% of the participants in the study group, the number of births was linked to one or two deliveries. As a result, the number of pregnancies and deliveries between the two groups did not differ statistically significantly.

With regard to abortion, 89.5% of the study group and 92.5% of the control group respectively never experienced an abortion. Additionally, the study group’s mean score for the length of the present pregnancy was 36.4 ± 1.2, while the group under control was 33.8 ± 3.5. The two groups’ mean scores for current pregnancy duration differed statistically significantly, actually.

The internal locus of control on health (IHLC) mean scores between the studied groups before and after the intervention are presented in Table 3. Prior to the program intervention, the study group mean score was 11.320 ± 8.859, whereas the control group mean score was 12.826 ± 9.750. The difference between the studied groups under study did not meet statistical significance (*p* = 0.096). Additionally, after the intervention, a very statistically difference within the mean scores of the internal health locus of control (IHLC) was observed in both the study and control groups. The study group’s value was 22.373 ± 9.316, while the control group’s value was 20.866 ± 9.969, with a *p*-value of 0.000.

The external powerful others (EPHLC) mean scores for both groups pre and post the intervention are presented in Table 4. Preprogram intervention, there was a non-highly statistically difference (*p* = 0.317) between the studied groups, with 15.226 ± 9.878 for the study group and 17.493 ± 10.319 for the control group. Additionally, post program intervention, a highly statistically difference in mean scores of external powerful others (EPHLC) was seen between both groups. The study group’s score was 24.866 ± 7.323, while the control group’s score was 23.640 ± 8.105, with a *p*-value of 0.000.

The external Chance (ECHLC) mean scores for the groups under study are compared between pre and post the intervention in Table 5. Prior to the intervention, the study group’s results were 11.453 ± 9.587, while the control group’s were 10.386 ± 8.635, indicating none statistically difference between both groups (*p* = 0.132). Moreover, after the intervention, the study and control groups’ external Chance (ECHLC) mean scores showed a non-highly significant difference. The experimental group’s value was 21.013 ± 9.047, while the control group’s was 19.946 ± 10.628 (*p* = 0.102).

**Fig. 2:**
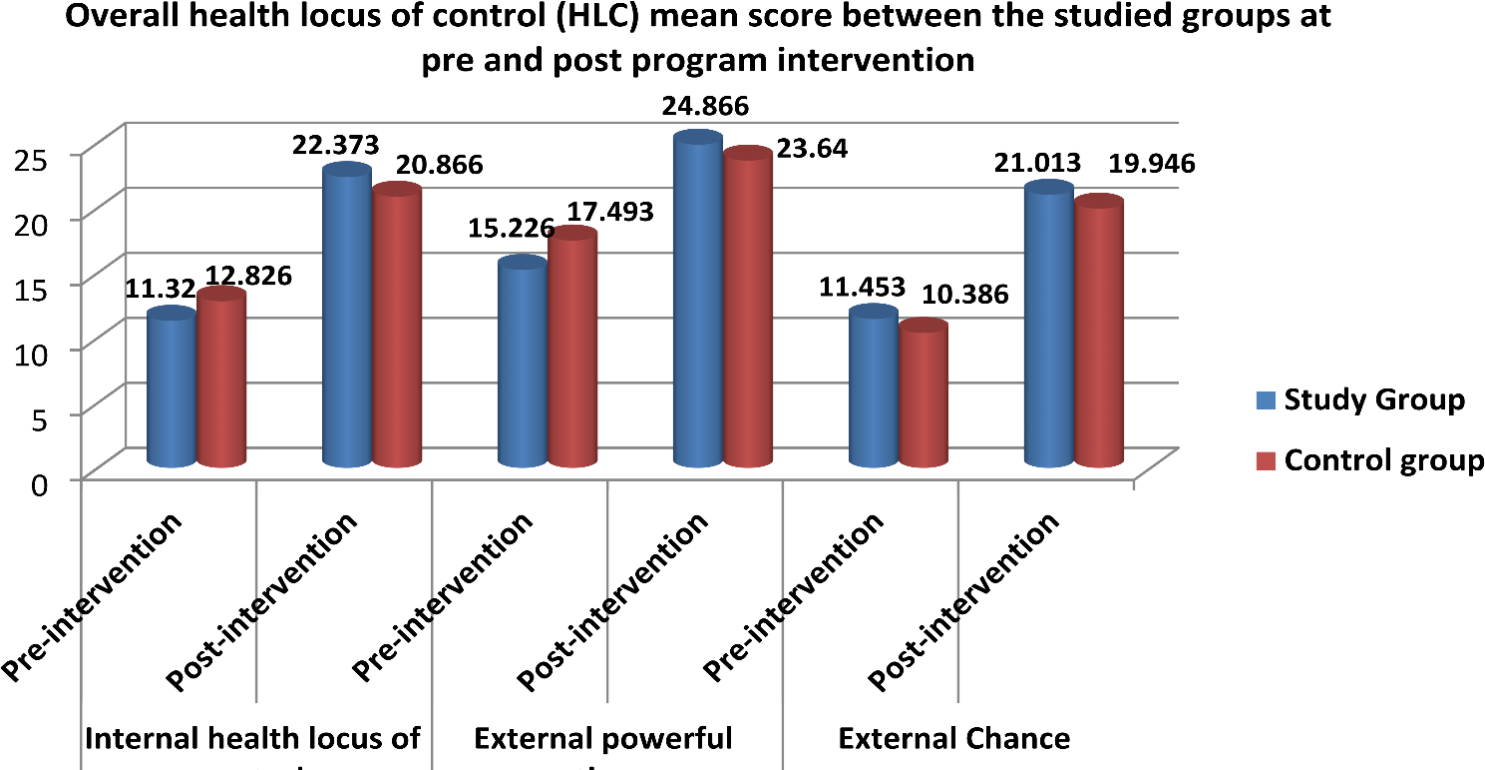
Overall health locus of control (HLC) mean scores dimension between the studied groups at pre and post program intervention

Figure 2 shows the mean scores for each locus of control on health (HLC) dimension for the studied groups both pre and post program intervention. When compared to the control group, which does not get nursing intervention educational sessions, it were shown that preeclamptic women in the study group who attended had a greater locus of control on health (internal, external powerful others, and external chance).

Differences of self-efficacy mean ratings for the studied groups pre and post program intervention are shown in Table 6. Prior to the intervention, there was highly statistically difference (*p* = 0.000) between the both groups, with 3.106 ± 1.236 for the study group and 3.533 ± 0.920 for the control group. Post the program intervention, a highly statistically difference of mean self-efficacy scores between both groups was also found. The study group’s value was 15.640 ± 5.116, while the control group’s value was 12.080 ± 6.803 (*p* = 0.000).

**Fig. 3:**
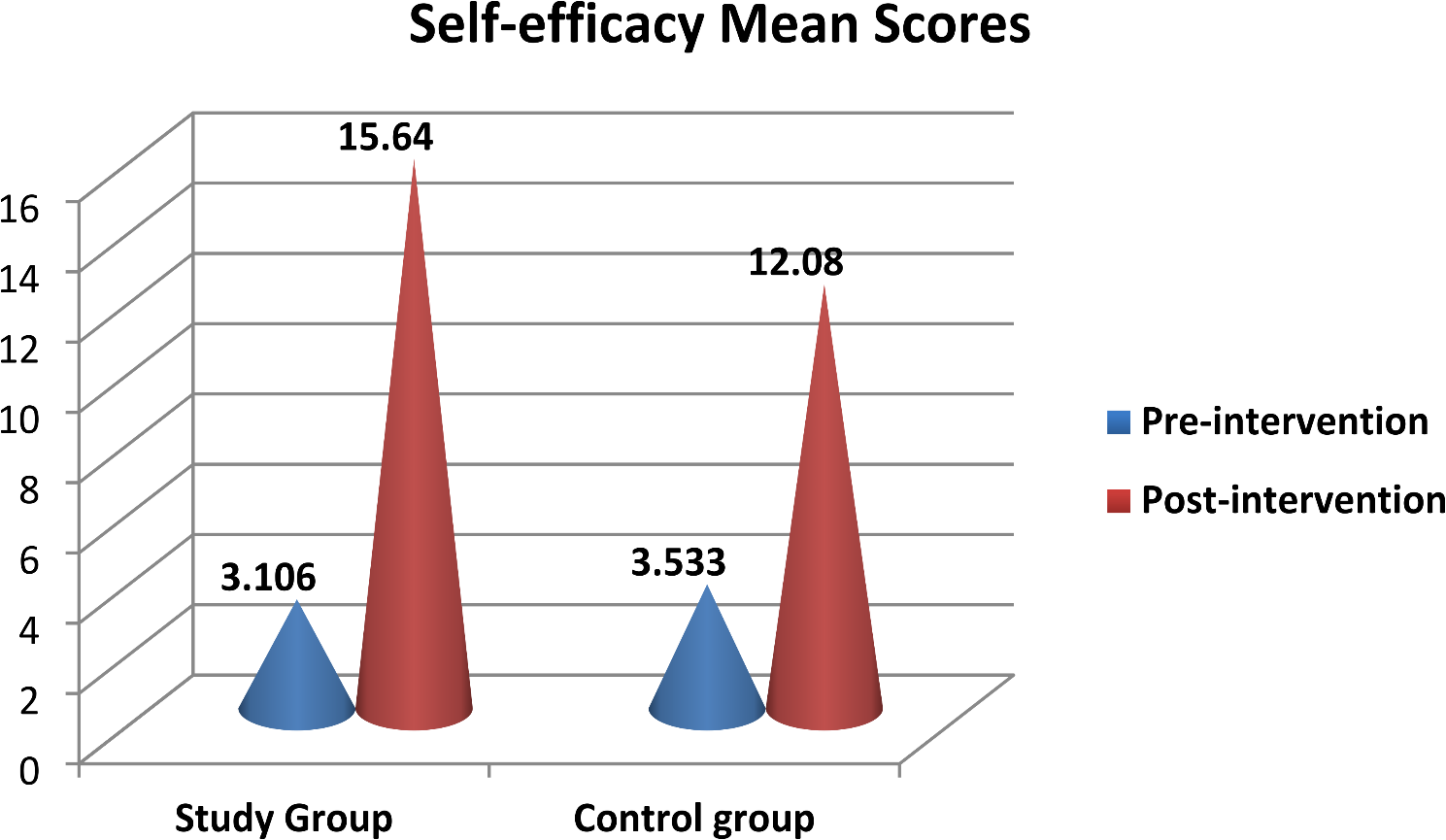
Overall self-efficacy mean scores between the studied groups at pre and post program intervention

Figure 3 shows that the study group’s mean self-efficacy score improved from 3.106 ± 1.236 pre-intervention to 15.640 ± 5.116 after the program intervention. The control group, mean self efficacy scores elevated slightly from 3.533 ± 0.920 prior to the intervention to 12.080 ± 6.803 following it.

**Table 1:** Frequency and percentage distribution of the studied groups regarding personal characteristics

Personal characteristics	Study group (*n* = 75)		Control group (*n* = 75)		Significance
	No.	%	No.	%	
**Age (years)**					t = 2.168 *P* = 0.036*
20–29	57	74.0	32	43.0	
30–35	20	26.0	43	57.0	
Range	20–35		20–35		
Mean ± SD	27.2 ± 5.8		31.6 ± 6.8		
**Educational level**					^**MC**^*P*=0.783
Write and read	4	5.0	4	5.0	
Primary	6	8.0	6	8.0	
Preparatory	10	13.0	14	19.0	
Diploma	39	52.0	38	51.0	
University	16	22.0	13	17.0	
**Employment**					X^2^ = 0.176 *P* = 0.675
House wife	43	57.0	46	62.0	
Employed	32	43.0	29	38.0	
**Family monthly income (LE)**					t = 0.65 *P* = 0.527
1000-<2000	5	7.0	5	7.0	
2000–3000	28	37.0	28	37.0	
> 3000	42	56.0	42	56.0	
Range	1000–3750		1000–3500		
Mean ± SD	2647.8 ± 537.5		2755.9 ± 513.4		
**Crowding index**					Z = 0.0 *P* = 1.0
Range	1.0–2.0		0.7-2.0		
Mean ± SD	1.4 ± 0.3		1.4 ± 0.3		

T: t-test, Z: Mann Whitney test, *significant at *P* ≤ 0.05, ^MC^P: Monte Carlo test, X^2^: Chi-Square test

**Table 2:** Distribution of the studied groups regarding their obstetric history

Obstetric history	Study group (*n* = 75)		Control group (*n* = 75)		Test	*P* value
	No	%	No	%		
**Number of gravida**:					Z = 1.214	0.226
**1**	36	48	38	51		
**2–3**	38	51	24	32		
**4**	1	1	13	17		
Range	1–3		1–4			
Mean ± SD	2.4 ± 1.1		1.8 ± 0.5			
**Para**:					Z = 1.168	0.243
**Prime**	37	49	38	51		
**1–2**	38	51	24	32		
**3**	0	0	13	17		
Mean ± SD	1.3 ± 1.2		0.9 ± 0.5			
**The total number of abortions**					Z = 1.411	0.457
**Yes**	8	10.5	6	7.5		
**No**	67	89.5	69	92.5		
**Weeks of the present pregnancy**:					t = 3.95	0.001*
Mean ± SD	36.4 ± 1.2		33.8 ± 3.5			

T: t-test, Z: Mann Whitney test, *significant at *P* ≤ 0.05

**Table 3:** Differences in the mean scores of internal health locus of control (IHLC) between the studied groups at pre and post program intervention

Groups	Study Group (*n* = 75) Mean ± SD	Control group (*n* = 75) Mean ± SD	Mean difference [95% CI]	T test (*P*)
Pre-intervention	11.320 ± 8.859	12.826 ± 9.750	5.720	20.796 (0.096)
Post-intervention (after one month)	22.373 ± 9.316	20.866 ± 9.969	22.373	22.002 (0.000) *

**Table 4:** Comparison of external powerful others (EPHLC) mean scores between the studied groups at pre and post program intervention

Groups	Study Group (*n* = 75) Mean ± SD	Control group (*n* = 75) Mean ± SD	Mean difference [95% CI]	T test (*P*)
Pre-intervention	15.226 ± 9.878	17.493 ± 10.319	6.000	21.166 (0.317)
Post-intervention (after one month)	24.866 ± 7.323	23.640 ± 8.105	24.866	29.406 (0.000) *

**Table 5:** Differences in the mean scores of external chance (ECHLC) between the studied groups at pre and post intervention

Groups	Study Group (*n* = 75) Mean ± SD	Control group (*n* = 75) Mean ± SD	Mean difference [95% CI]	T test (*P*)
Pre-intervention	11.453 ± 9.587	10.386 ± 8.635	5.400	17.378 (0.132)
Post-intervention (after one month)	21.013 ± 9.047	19.946 ± 10.628	21.013	(0.102) 22.997

**Table 6:** Differences of self efficacy mean scores in the studied groups at pre and post program intervention

Groups	Study Group (*n* = 75) Mean ± SD	Control group (*n* = 75) Mean ± SD	Mean difference [95% CI]	T test (*P*)
Pre-intervention	3.106 ± 1.236	3.533 ± 0.920	3.319	16.649(0.000) *
Post-intervention (after one month)	15.640 ± 5.116	12.080 ± 6.803	13.360	26.472(0.000) *

## Discussion

For preeclamptic women, adherence to medication is a powerful determinant in determining how well the antihypertensive regimen works. The process is intricate and involves a woman’s understanding and perception of her sickness, her drive to control it, her self-assurance in her capacity to carry out illness-management techniques, her expectations for the course of treatment, and her awareness of the negative effects of non-compliance. Consequently, a complicated set of self-management behaviors aimed at managing the disease are expected of preeclamptic women [21]. The knowledge and adherence to the antihypertensive regimen among these women typically varies greatly. In this regard, it has been acknowledged that the locus of control is a significant domain that influences preeclamptic women’s understanding and adherence to the treatment plan. In fact, investigate how any action made to prevent health issues is mediated by locus of control. It has a positive correlation with accomplishments, changing attitudes, and conformity [17]. Therefore, this study assessed the effectiveness of nursing intervention session on health locus of control and self efficacy for women with preeclampsia.

According to the current study, internal mean score of locus of control over health will be higher for preeclamptic women who attend nurse intervention sessions than for the control group. As a result, there is non-statistically difference between both groups prior to intervention, but there was a highly statistically difference between both group post intervention where (*p* < 0.001); the study group that received program intervention showed when compared to the control group, which does not attend program intervention sessions, there was a substantial improvement in the mean score of internal locus of control following the program intervention. This results due to the fact that the program intervention sessions having a favorable effect on their locus of control ratings for internal health by giving the study group more control over their life and a greater sense of accountability for managing their illness and coming up with practical control strategies. Additionally, about 50% of the mothers in the study group had one or two pregnancies and might had some experience.

This result was in line with Pieczykolan et al. [17]. who mentioned that pregnant mothers have a locus of health control, which is significant when coping with challenging circumstances like a high-risk pregnancy. The findings show that the internal control scale had the highest values across all locus of control on health dimensions. In this research, the average health control level in the experimental group was 26.25 in the internal influence dimension, which was statistically higher than the level in the control group, which was 24.61. Furthermore, in contrast to those who have a limited sense of control over their health, those with a greater sense of internal health control are more likely to display behaviors that promote health. That being said, complying with healthcare recommendations and promoting health actions are less likely in the presence of a high degree of health control in relation to chance.

Moreover, according to Zaky [22], individuals who possess an internal HLC think they have the power to control their own health and are more likely to follow treatment plans because they accept responsibility for their actions and make decisions free from outside influence. However, the internality believes that they have the ability to safeguard their own health.

In terms of external powerful others of locus control over health, the present study postulated that preeclamptic women who take part in program intervention sessions will have score higher on average better than the control group. The current result indicates that, following the program intervention, there was a statistically difference mean scores for locus of control over external health between the studied groups. This conclusion could arise from the preeclamptic women’s attempts to participate in practices that promote health in this study. They believe that influential people, including doctors, regulate their expectations.

This finding was in agreement with the findings of Pieczykolan et al. [17]., who reported that the study group’s state of a healthy management in the influence of others dimension was 22.15, but the control group’s level was 20.99. The differences that were found were significant statistically. However, this trend among pregnant women is a desirable one, as the conviction that one must take responsibility for their personal well-being will influence actions that promote healthy behaviors, like adhering to a low-carbohydrate diet, conducting controls as prescribed by a physician, and routinely monitoring blood pressure. These actions will lower the likelihood of various pregnancy complications in the future.

Based on the current research, it was predicted that preeclamptic women who attended program intervention sessions would have greater mean scores for external probability of health locus control than the control group. Regarding the health locus control mean scores over external chance at pre and post program intervention, the current finding shows none statistically significant difference between both groups. This results could be due to that the preeclamptic women in the study believing that others, such family members, manage random expectations like fate or luck. Not only do they think they have no influence over their lives, but they also typically lack initiative and put in little effort for recognizing their own potential.

This finding was supported by Pieczykolan et al. [17]. provided confirmation for this finding, stating that within the influence of chance dimension, the control group’s level of health control was 19.24, but the result for the health study group was 20.36. The data showed no discernible statistically significant variation.

Moreover, Fardaza et al. [23] found that the external chance mean score of health locus control prior to program intervention did not show a statistically difference between both groups. Following nursing intervention sessions, the current study mentioned that both groups’ external chance health locus of control had improved, with a statistically difference between both groups. This discrepancy could be the result of variations in the questionnaire used to collect the data. Thus, there is some support for the first hypothesis.

The current study made the hypothesis that preeclamptic women in study group would score higher on self-efficacy tests than the control group if they attended nurse intervention sessions. According to the current data, there were statistically difference (*p* = 0.000) in the study group’s self-efficacy levels following the program intervention compared to the control group. Between before and after program intervention periods in two groups, there was a statistically significant increase in self-efficacy, and there was a statistically difference between both groups. Furthermore, according to the results, the program intervention has been more effective for preeclamptic women when it gives all expectant mothers a sense of empowerment and encourages them to adhere to health recommendations such as self-monitoring blood pressure, verifying for proteinuria, scheduling regular prenatal visits, staying in bed, and seeking medical attention that may improve the outcome of their pregnancy.

The current results were consistent with a study by Pieczykolan et al. [17]., which noted that the study group’s average level of self-efficacy was statistically greater than the group’s control, which consisted of expecting mothers whose pregnancies have been uncomplicated (29.62) and was throughout the upper range of the mean reference values at 28.47. Additionally, according to Mohebbi, Tol, Sadeghi, Mohtarami, and Shamshiri [24], a woman is more motivated to change her health-related behaviors when she has a high degree of self-efficacy and confidence, as well as sufficient understanding about the illness and how it affects the unborn child and pregnancy.

Additionally, Eshghi Motlagh, Babazadeh, Akhlaghi, & Esmaily [25] confirmed the current findings. According to their findings, women’s perception of self-efficacy was raised by educational techniques like verbal persuasion and behavioral pattern modification, which are within the framework of Bandura’s Self-Efficacy Theory (SET). It was widely assumed that women who verbally believed they had certain abilities and could try harder to overcome challenges could do so because they felt more confident in their abilities. In the end, the study’s findings supported the acceptance of the self-efficacy research assumptions.

## Conclusion

According to the current study’s findings which concluded that, after participating in nursing intervention sessions, pregnant women with preeclampsia had higher mean scores for internal and external powerful others in locus control over health, and self-efficacy than the control group, who didn’t not take part in nurse intervention sessions with a significantly difference between the studied groups. Additionally, the study group’s mean scores for external chance of health locus control were greater than those of the control group’s, with none discernible difference between the groups.

### Limitations of the study

In addition to organizing challenges, there was no permanent location for the educational sessions.

### Recommendation

This study recommended that:

- Educating women on the effects of preeclampsia and how locus of control and self-efficacy can help reduce difficulties for mothers and newborns when a woman has preeclampsia.
- All women at risk of preeclampsia should receive psychological support and education regarding the significance of maintaining healthy lifestyle decisions and actions in the management of their condition.
- Offer health education initiatives for all women at risk of preeclampsia in order to enhance their locus of control over their health and sense of self-efficacy.

## Data Availability

Because of confidentiality considerations, the current study’s data and materials cannot be made public. However, they can be obtained from the appropriate author upon reasonable request.
